# Nomogram and recursive partitioning analysis to predict overall survival in patients with stage IIB-III thoracic esophageal squamous cell carcinoma after esophagectomy

**DOI:** 10.18632/oncotarget.10904

**Published:** 2016-07-28

**Authors:** Shufei Yu, Wencheng Zhang, Wenjie Ni, Zefen Xiao, Xin Wang, Zongmei Zhou, Qinfu Feng, Dongfu Chen, Jun Liang, Dekang Fang, Yousheng Mao, Shugeng Gao, Yexiong Li, Jie He

**Affiliations:** ^1^ Department of Radiation Oncology, Cancer Institute (Hospital), Chinese Academy of Medical Sciences and Peking Union Medical College, Beijing 100021, China; ^2^ Department of Radiation Oncology, Tianjing Medical University Cancer Institute and Hospital, National Clinical Research Center of Cancer, Tianjin 300000, China; ^3^ Department of Thoracic Surgery, Cancer Institute (Hospital), Chinese Academy of Medical Sciences, Peking Union Medical College, Beijing 100021, China; ^4^ Department of Oncology, Beijing Chao-yang Hospital, Beijing 100000, China

**Keywords:** esophageal carcinoma, esophagectomy, overall survival, nomogram, recursive partitioning analysis

## Abstract

We have developed statistical models for predicting survival in patients with stage IIB–III thoracic esophageal squamous cell carcinoma (ESCC) and assessing the efficacy of adjuvant treatment. From a retrospective review of 3,636 patients, we created a database of 1,004 patients with stage IIB–III thoracic ESCC who underwent esophagectomy with or without postoperative radiation. Using a multivariate Cox regression model, we assessed the prognostic impact of clinical and histological factors on overall survival (OS). Logistic analysis was performed to identify factors to include in a recursive partitioning analysis (RPA) to predict 5-year OS. The nomogram was evaluated internally based on the concordance index (C-index) and a calibration plot. The median survival time in the training dataset was 30.9 months, and the 5-year survival rate was 33.9%. T stage, differentiated grade, adjuvant treatment, tumor location, lymph node metastatic ratio (LNMR), and the presence of vascular carcinomatous thrombi were statistically significant predictors of 5-year OS. The C-index of the nomogram was 0.70 (95% CI 0.67–0.73). RPA resulted in a three-class stratification: class 1, LNMR ≤ 0.15 with adjuvant treatment; class 2, LNMR ≤ 0.15 without adjuvant treatment and LNMR > 0.15 with adjuvant treatment; and class 3, LNMR > 0.15 without adjuvant treatment. The three classes were statistically significant for OS (*P* < 0.001). Thus, the nomogram and RPA models predicted the prognosis of stage IIB–III ESCC patients and could be used in decision-making and clinical trials.

## INTRODUCTION

Esophageal carcinoma is the third most prevalent cancer and the fourth leading cause of cancer-related deaths in China [1]. Approximately 477,900 Chinese patients were diagnosed with esophageal cancer (both esophageal squamous cell carcinoma and esophageal adenocarcinoma) in 2015 and approximately 375,000 patients died from the disease. Peyre et al. [2] found that the number of involved lymph nodes was positively correlated with systemic disease (16–93%). The 7th edition of the Union for International Cancer Control/American Joint Cancer Committee (UICC/AJCC) included a fundamental change in the N classification from site-dependent to numerically based staging. The 5-year OS ranged from 8–45% for patients with stage IIB–III esophageal carcinoma [3]. The 7th edition of the UICC/AJCC prognostic grouping [4, 5] did not perform as well for stage IIB–III patients as for early stage patients. Reeh et al. reported that the 7th edition TNM staging system poorly discriminated between stages IIIA and IIIB (*p* = 0.672), and stages IIIC and IV (*p* = 0.799) [6]. The new AJCC prognostic groupings for early stage patients include the histologic type [7], grade [8–10], and location of the tumor in addition to the TNM classification [3]. We hypothesized that an improved prognostic grouping could be achieved by including additional parameters associated with survival.

Nomograms can be used to quantify risk by incorporating factors that impact prognosis [11, 12]. By creating an intuitive graph of a statistically predictive model, a nomogram gives rise to a numerical probability of a clinical event (e.g. OS). Nomograms can generate more accurate predictions than the traditional TNM staging system [13–18]. However, few nomograms exist that can predict the long-term survival of patients with locally advanced esophageal squamous cell carcinoma (ESCC) post-esophagectomy. In this study, we aimed to build and validate a nomogram for locally advanced ESCC, which combined known clinicopathological variables, using data from the Chinese Academy of Medical Sciences.

## RESULTS

**Figure 1:**
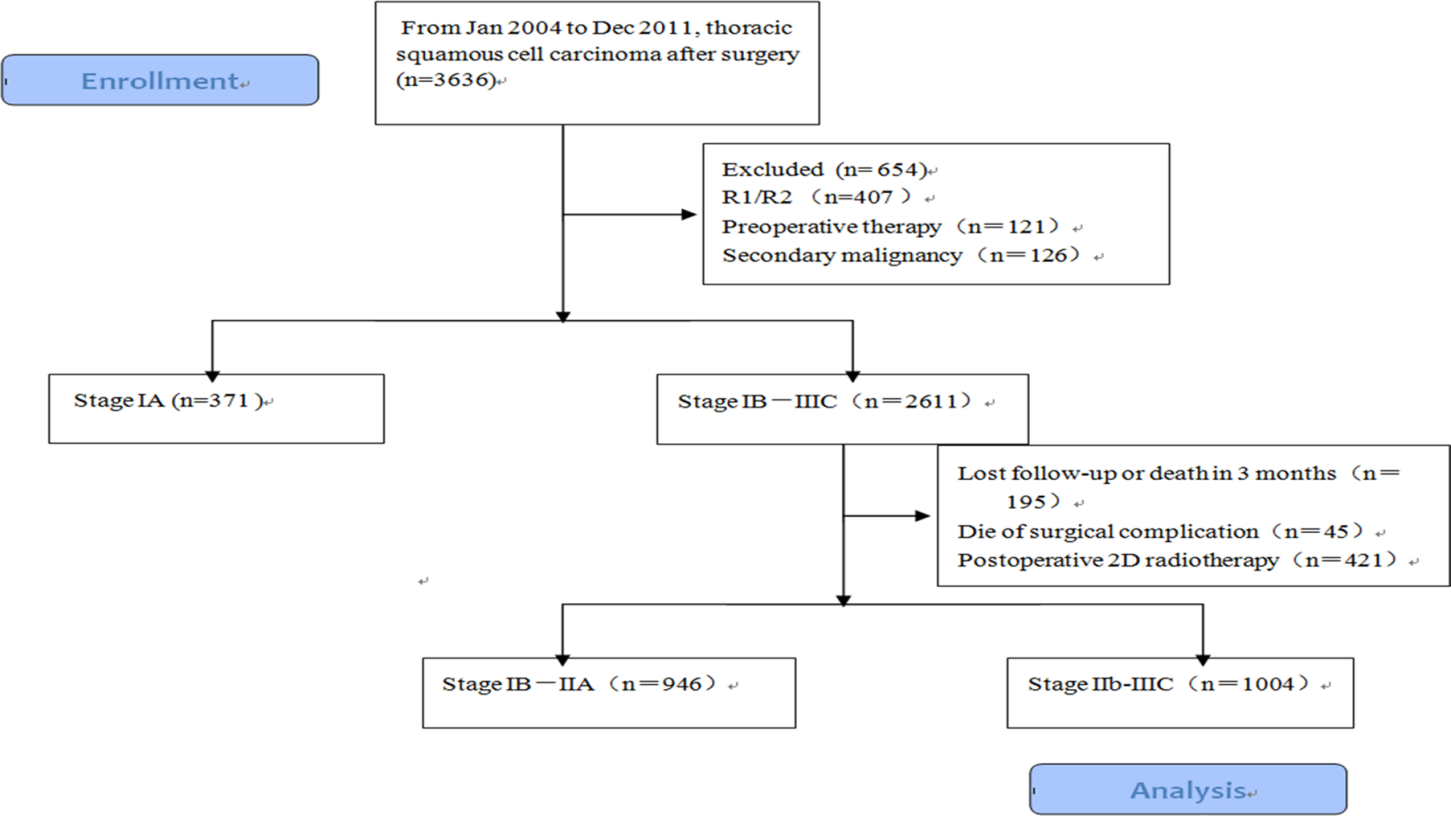
Flow diagram of the patients received radical surgery in Chinese Academy of Medical Sciences, 1004 patients in stage IIb-III were enrolled

Demographic and characteristics of patients are shown in Table 1 and Figure 1. The median follow-up in the training cohort was 67.5 months (95% CI 64.8–70.7 months). The median survival and 5-year OS rates in the training cohort were 30.9 months (95% CI 28.1–33.7 months) and 34.2%, respectively. The 5-year OS rates were 48.5%, 38.2%, 23.2%, and 23.3% for patients with stage IIB, IIIA, IIIB, and IIIC disease, respectively (*p* < 0.001). However, there was no statistically significant difference between patients with stage IIIB or IIIC disease (*p* = 0.527).

**Table 1:** Patients' characteristics

Characteristics	Number of patients	(%)
**Sex**		
Male	847	84.4
Female	157	15.6
**Age**		
≤ 60	564	56.2
> 60	440	43.8
**T stage(7th UICC)**		
T1	103	7.3
T2	147	14.6
T3	683	68.0
T4a	101	10.1
LNMR	20	2.0
0–0.073	395	39.3
0.073–0.15	297	29.6
> 0.15	292	29.1
**Tumor Location**		
Upper third	81	8.1
Middle third	471	46.9
Lower third	452	45.0
**Treatment**		
Surgery	490	48.8
Surgery + adjuvant	514	51.2

### Nomogram model development and validation

**Figure 2:**
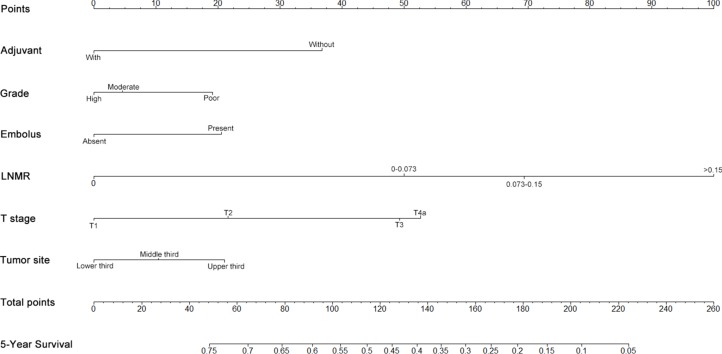
Nomogram to predict 5-year survival in patients with stage IIb−III ESCC To use the nomogram, the value attributed to an individual patient is located on each variable, and a line is then drawn downwards to the survival axis to determine the 5-year OS likehood.

**Figure 3:**
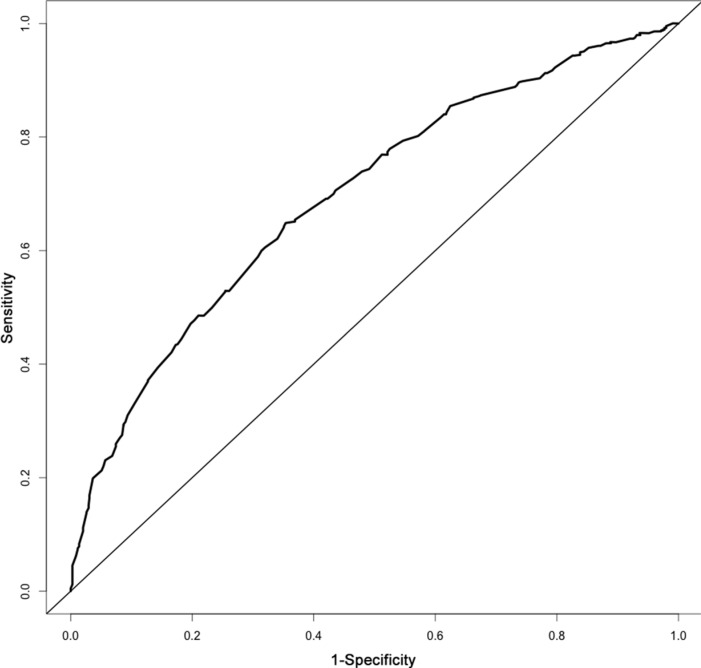
The area under the receiver operating characteristic (ROC) curve of the nomogram for stage IIb-III ESCC was 0.70

**Figure 4:**
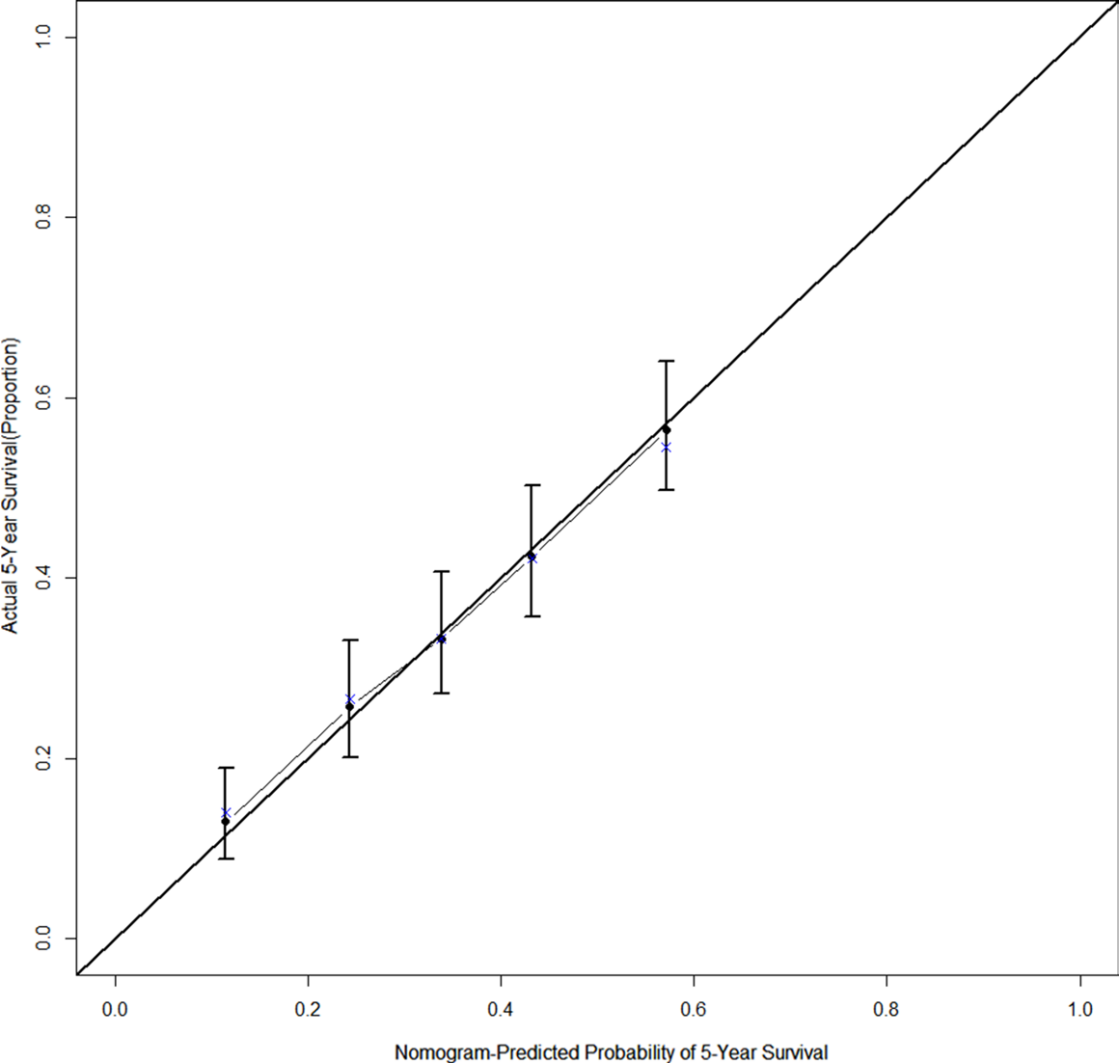
The calibration curve for predicting 5-year survival after esophagectomy in stage IIb−III thoracic ESCC patients, the nomogram-predicted probability of OS is plotted on the x axis; the actual observed OS is plotted on the y axis

**Figure 5:**
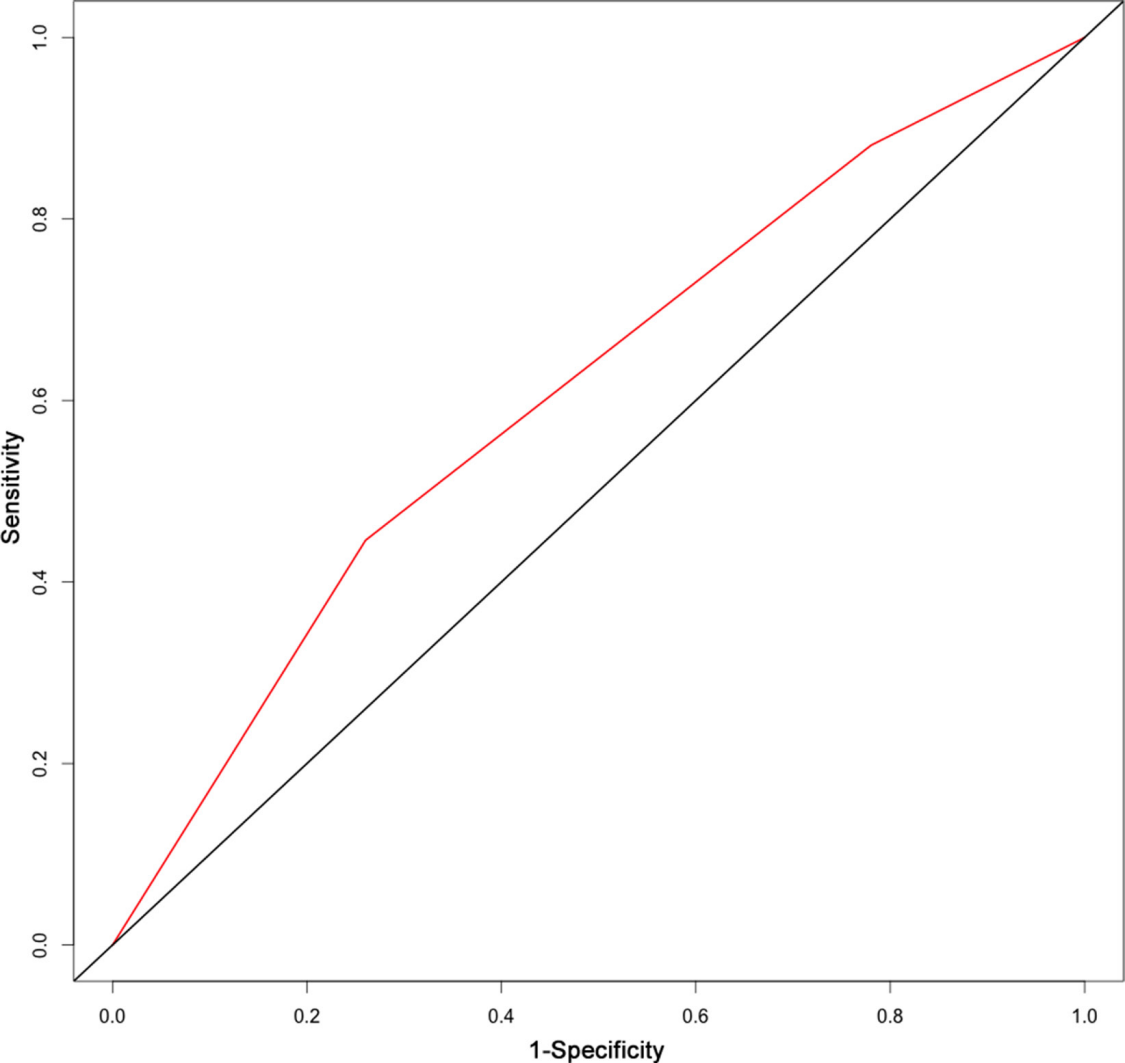
The area under the receiver operating characteristic (ROC) curve of 7th UICC staging system for stage IIb-III ESCC was 0.61

The results from a multivariate analysis of the training dataset are shown in Table 2. Based on these data, a nomogram was developed to predict 5-year OS (Figure 2), which incorporated adjuvant therapy, differentiated grade, the presence of vascular carcinomatous thrombi embolus, T stage (7th UICC stage), tumor location (6^th^ UICC stage), and the lymph node metastatic ratio (LNMR). The predictive discrimination for 5-year survival was measured by the concordance index (C-index). The C-index of the nomogram was 0.70 (95%CI 0.67–0.73) (Figure 3), which demonstrated good accuracy in the training cohort. However, the C-index of 7th UICC staging system was 0.61 (95% CI 0.58–0.64) (Figure 4) in the training dataset, which was lower than that of the nomogram in this cohort. We next used calibration plots to assess whether the nomogram estimated risk was in consistent with the observed risk. Indeed, calibration plots confirmed a strong correlation between the observed and predicted probability of 5-year OS for the entire cohort (Figure 5).

**Table 2:** Multivariate analysis of patients in primary cohort

Variables	HR	95%CI	*P*
**Grade**			0.045
High	Ref.		
Moderate	1.065	0.826–1.374	0.625
Low	1.291	0.989–1.685	0.061
**Embolus**			0.004
Absent	Ref.		
Present	1.329	1.094–1.602	0.004
**Tumor site**			0.087
Upper third	Ref.		
Mid third	0.868	0.652–1.156	0.033
Lower third	0.755	0.564–1.011	0.059
**LNMR**			0.000
0	Ref.		
0–0.073	1.948	0.928–4.092	0.078
0.074–0.15	2.513	1.192–5.195	0.015
> 0.15	3.773	1.795–7.933	0.000
**T stage**			0.000
T1	Ref		
T2	1.334	0.899–1.980	0.152
T3	1.931	1.365–2.731	0.000
T4	2.013	1.307–3.101	0.001
**Treatment**			0.000
Surgery	Ref.		
Surgery + adjuvant	0.615	0.523–0.722	0.000

**Figure 6:**
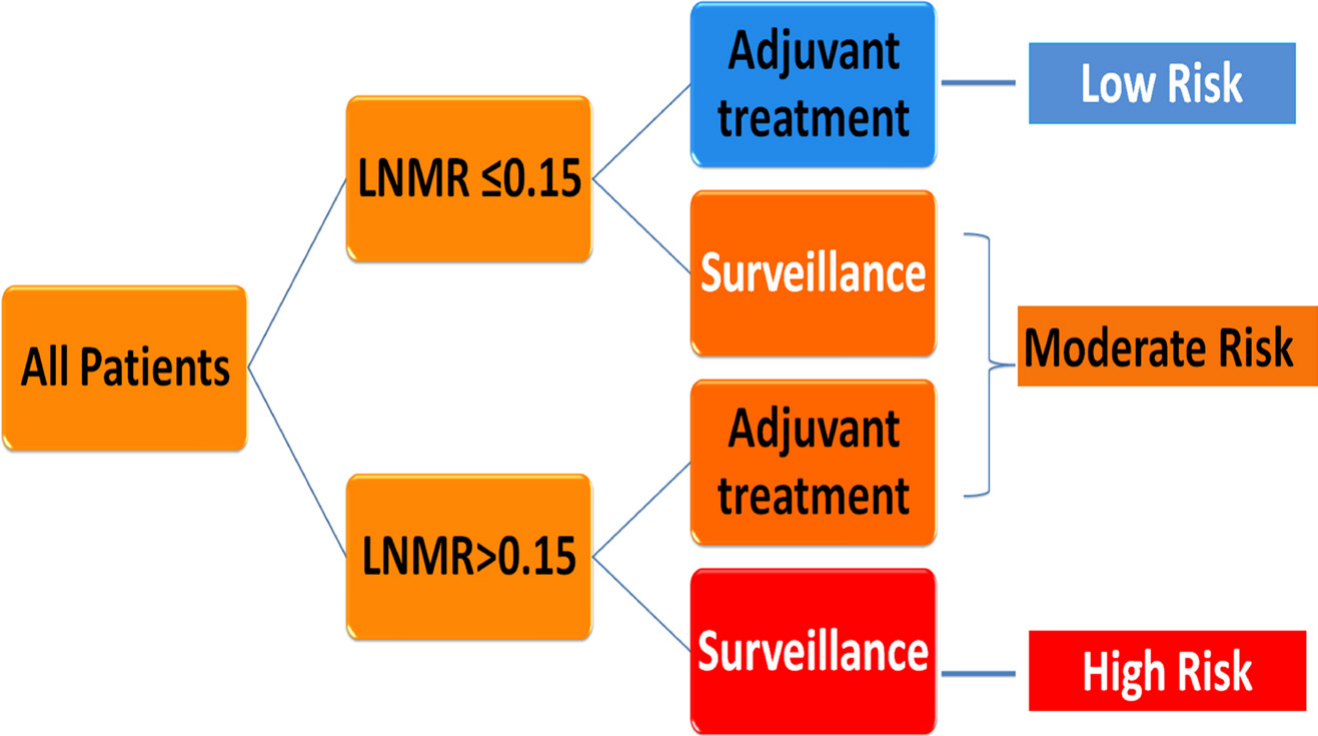
Decision tree constructed by recursive partitioning analysis for patients with stage IIb-III ESCC

**Figure 7:**
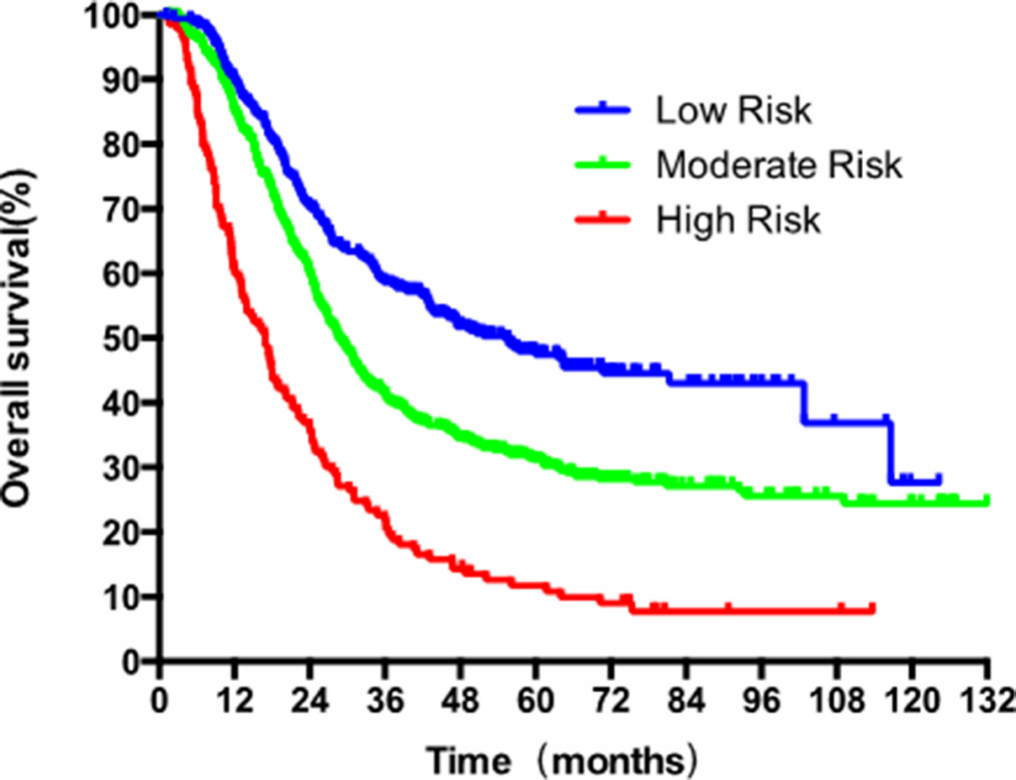
Survival analysis with Kaplan-Meier plot based on the three risk categories generated by recursive partitioning analysis for 5-year OS

The significant factors for survival identified by multivariate cox regression, adjuvant therapy, differentiated grade, the presence of embolus, T stage (7th UICC stage), and the LNMR, were entered the model. LNMR and adjuvant therapy provided a tree with three nodes, separating patients into four classes; class 1: LNMR ≤ 0.15 with adjuvant treatment; class 2: LNMR ≤ 0.15 without adjuvant treatment; class 3: LNMR > 0.15 with adjuvant treatment; class 4: LNMR > 0.15 without adjuvant treatment. Differentiated grade, the presence of vascular carcinomatous thrombi embolus, T stage (7th UICC stage) did not contribute significantly in the modeling and were omitted from the tree. No statistical difference in OS was shown between class 2 and class 3 (*p* = 0.167), using log-rank test. Therefore, class 2 and 3 were collapsed into a single class. In the final RPA model for OS (Figure 6), class 1 was considered as low-risk group (5-year OS rate 47.4%), class 2 and 3 were considered as intermediate risk group (5-year OS rate 31.1%) and class 4 was considered as high risk group (5-year OS rate 11.7%). There were significant differences in OS (Figure 7) between risk groups on univariate Cox analysis (Hazard Ratio [HR] = 1.753, 95% CI 1.558–1.973; *p* < 0.001).

## DISCUSSION

The 7th edition of the AJCC TNM staging system, which was based on risk-adjusted random forest analyses of data from the Worldwide Esophageal Cancer Collaboration (13 institutions and 4,627 patients treated with primary esophagectomy alone) introduced new number-based N subgroups [19]. However, poor discrimination was observed in the Kaplan-Meier survival curves for each subgroup of patients. A previous report indicated that the 7th edition staging system could not discriminate between stage IIA versus IIB, IIIA versus IIIB, or IIIC versus IV [3]. In our patient cohort, the 7th edition criteria could not distinguish between stage IIIB versus IIIC (*p* = 0.537). Thus, improvements in the prognostic model and risk group are warranted.

A robust model consisting of a nomogram that can be used to precisely estimate stage IIB–III ESCC patient survival has not been developed previously. Additionally, RPA for risk stratification, which could improve the clinical management of ESCC patients, has not been performed. Therefore, we developed a postoperative model to predict long-term survival and guide the clinical management of this patient population. The dataset was obtained from leading institution in China, which represents standard and advanced oncology care. Thus, the model is predicted to be easily generalizable.

On univariable and multivariable analyses, T stage, LNMR, adjuvant treatment, differentiated grade, and the presence of an vascular carcinomatous thrombi embolus were independent prognostic factors for ESCC. Differentiated grade and tumor location are considered prognostic factors for early-stage ESCC in the AJCC staging system [20], but not for locally advanced disease. Here, we defined tumor location based on the 6^th^ edition of the UICC staging system [21] and incorporated it into the model. Tumor location was documented during surgery for most patients. Previous studies have demonstrated that the N group was a good prognostic factor for survival [2, 22–32]. Lymphatic metastasis in esophageal carcinoma is widespread dissemination from lower cervical and supraclavicular region to the celiac lymph node basins. Number of harvested lymph nodes may associate with the longer survival for the probabilities of missing a positive lymph node and erroneously classified with an earlier stage cancer decrease. Currently, there is no international consensus as to the minimum number of lymph nodes that should be examined. It has been proposed that at least 12 lymph nodes be removed for pathological analysis [22]. Peyre et al. [32] reported that the removal of more than 23 lymph nodes was a favorable prognostic factor. However, Altorki et al. [33] found that the removal of 16–30 lymph nodes in two-field lymphadenectomy did not improve survival. Both Schwarz et al. [34] and Groth et al. [35] suggested that more than 30 lymph nodes should be removed during radical surgery for esophageal carcinoma, which was based on an analysis of a large number of samples. In the present study, we hypothesized that the LNMR (the ratio of positive nodes and removed lymph nodes) could accurately predict survival in both adequately and inadequately staged patients. LNMR varies based on the segment of the esophagus that is involved and may be correlated with the operative approach, range, and number of lymph nodes examined. The LNMR has been suggested as an important prognostic factor in many cancers [33, 36–44]. Using ROC curve, we determined that a LNMR of 0.073 was the optimal cut-off point, which resulted in a maximum Youden's index value. To make the distribution of patients among the groups balanced, a second cut-off value was 0.150. Adjuvant treatment was also incorporated into the model because subgroup analyses of prospective randomized clinical trials as well as retrospective analyses have indicated that it can improve OS [45–55]. The predictive accuracy of the nomogram increased when non-TNM factors were included. The RPA-based risk group was distinctive for each group in the training dataset (*p* < 0.001).

Validation of the nomogram was essential to avoid over-fitting of the model and determine generalizability. In this study, calibration plots showed optimal agreement between predictions and actual observations, which provided evidence for the repeatability and reliability of the nomogram. Discrimination of the nomogram was revealed by the higher C-index of the nomogram compared to the TNM staging system (C-index 0.61) in the training cohort. We found that the 7th edition AJCC staging system was not distinctive for stage IIIB versus IIIC in the training dataset (*p* = 0.537).

To the best of our knowledge, this is the first nomogram and RPA model for predicting the survival of locally advanced ESCC patients based on a large sample size and a long-term follow-up. Both physicians and patients could perform an individualized survival prediction after radical surgery through this easy-to-use scoring system. The identification of subgroups of patients at different risks for poor survival might impact treatment. We believe that the established nomogram and RPA models are more accurate prognostic models that the TNM staging system. Nonetheless, the models are limited by the retrospective nature of the data collection, which prevented the incorporation of several commonly recognized prognostic parameters (e.g. EGFR expression [56] and the circumferential resection margin [57]).

External validation is required for this model. Additional prospective studies including patients with planned follow-up, as well as the incorporation of several other factors are necessary to improve the model.

In conclusion, we have established and validated a novel model using a nomogram and RPA to predict ESCC patient survival and to stratify patients into different risk groups. This model can be used to make treatment decisions in patients with ESCC.

## MATERIALS AND METHODS

### Patient characteristics

We performed a retrospective review of a cohort consisting of 3,636 patients who were diagnosed with thoracic ESCC and underwent esophagectomy between January 1, 2004 and December 31, 2011 at the Chinese Academy of Medical Sciences (Figure. 1). All patients were restaged based on the 7th edition of the UICC/AJCC staging system. Patients who underwent radical surgery and were pathologically diagnosed with stage IIB−III disease were enrolled in the study. The exclusion criteria were the following: 1) preoperative treatment; 2) inadequate follow-up data; 3) pathological type other than squamous cell carcinoma; 4) diagnosis of a malignancy other than esophageal carcinoma. The remaining 1,004 patients comprised the training dataset (Table 1). All patients underwent radical esophagectomy (R0) with two- or three-field lymphadenectomy. There were 514 patients (51.0%) who did not receive any postoperative treatment and 490 (49.0%) who did receive adjuvant treatment. A total of 56 patients (5.6%) received adjuvant chemotherapy, 323 (32.2%) received adjuvant radiotherapy, and 135 (13.4%) received both chemotherapy and radiotherapy. Adjuvant therapy was initiated 4−6 weeks after surgery. Patients received either a taxane- or fluorouracil-based regimen. Radiotherapy was administered at a total dose of 50–60 Gy (1.8–2.0 Gy per fraction). This project was approved by our Institutional Review Board and conducted in accordance with the Declaration of Helsinki.

### Nomogram construction and recursive partitioning analysis

Factors anticipated to influence survival and variables that retained independent statistical significance in multivariate analyses were introduced into the nomogram. Variables that achieved significance at *p* < 0.1 in univariate analyses were incorporated into the multivariable analyses through a Cox regression model. Selection of the final model was performed using a backward step-down process. Nomogram validation included: 1) internal validation, in which the Harrell's C-index [58] was estimated by analyzing the area under the curve (AUC) of the receiver operating characteristic (ROC) curve to obtain an unbiased measure of the ability of the nomogram to discriminate between patients. The C-index ranged from 0.5−1.0, where 0.5 was indicative of random chance and 1.0 was indicative of perfect discrimination of the outcome using the model; 2) calibration, which was performed in order to examine how well the model-based predicted probability of survival agreed with the observed probabilities (in this calculation, 200 bootstrap resamples were used to generate the 95% CIs for the plot). Statistical analyses to identify independent prognostic factors were conducted using SPSS 20.0 for Windows (SPSS, Chicago, IL, USA). Based on the results of the multivariable analysis, a nomogram was formulated using the R software (http://www.r-project.org) with the survival ROC, Hmisc, and rms packages.

RPA provides a simple way to group patients into different categories. RPA divided patients at each step into two groups based on the covariate that provided maximum separation with respect to prognosis and accounted for interactions between factors [59]. This analysis was performed using the training dataset to predict the primary endpoint based on an inventory of eight factors. The R software with the rpart package was used for the analysis, where a minimum of 20 observations was required to split a node. This was followed by trimming of less important downstream branches. A minimum of 10 observations (default) was required for a terminal node. The clinical utility was enhanced by rounding the cut-off points to the nearest significant digit. The OS rates were compared between the RPA risk groups using log-rank tests. The primary endpoint was survival at the end of the 5-year period, which was calculated from the date of surgery to the date of either the last follow-up or death using the Kaplan-Meier method. Follow-up was calculated using the reverse Kaplan-Meier method. Patients who were alive as of November 10, 2015 were censored as of that date.
